# Antagonism between BRCA2 and FIGL1 regulates homologous recombination

**DOI:** 10.1093/nar/gkz225

**Published:** 2019-04-03

**Authors:** Rajeev Kumar, Marine Duhamel, Eve Coutant, Emna Ben-Nahia, Raphael Mercier

**Affiliations:** Institut Jean-Pierre Bourgin, INRA, AgroParisTech, CNRS, Université Paris-Saclay, 78000 Versailles, France

## Abstract

Homologous recombination (HR) maintains genome stability by promoting accurate DNA repair. Two recombinases, RAD51 and DMC1, are central to HR repair and form dynamic nucleoprotein filaments *in vivo* under tight regulation. However, the interplay between positive and negative regulators to control the dynamic assembly/disassembly of RAD51/DMC1 filaments in multicellular eukaryotes remains poorly characterized. Here, we report an antagonism between BRCA2, a well-studied positive mediator of RAD51/DMC1, and FIDGETIN-LIKE-1 (FIGL1), which we previously proposed as a negative regulator of RAD51/DMC1. Through forward genetic screen, we identified a mutation in one of the two Arabidopsis *BRCA2* paralogs that suppresses the meiotic phenotypes of *figl1*. Consistent with the antagonistic roles of BRCA2 and FIGL1, the *figl1* mutation in the *brca2* background restores RAD51/DMC1 focus formation and homologous chromosome interaction at meiosis, and RAD51 focus formation in somatic cells. This study shows that BRCA2 and FIGL1 have antagonistic effects on the dynamics of RAD51/DMC1-dependent DNA transactions to promote accurate HR repair.

## INTRODUCTION

Homologous recombination (HR) is critical for preserving genome integrity because it facilitates error-free DNA repair (1). In somatic cells, a dysfunctional HR process can lead to greater genomic instability and has been linked to the development of various types of cancer and genetic diseases in humans (2). In meiotic cells, HR is essential for generating crossovers (COs)—the reciprocal exchange of genetic material among homologous chromosomes. COs among homologs are crucial to establish the physical link required for the accurate segregation of homologous chromosomes (3). Therefore, errors or a lack of meiotic HR can cause sterility or chromosomal mis-segregation leading to aneuploidy diseases such as Down’s syndrome in humans (4). In addition, COs enhance genetic diversity in offspring by reshuffling the parental genomes in sexually reproducing organisms.

During meiosis, the HR repair process is initiated by the developmentally programmed formation of DNA double-strand breaks (DSBs) (5). Nucleolytic processing at break sites generates 3′ single-stranded DNA (ssDNA) overhangs. These ssDNA overhangs are used for homology searches, and subsequent strand invasion of the intact homologous template forms recombination intermediates wherein the invading strand serves as a primer for DNA synthesis (6). These intermediates can give rise to two types of repair products: CO with reciprocal exchanges among homologous chromosomes and non-crossovers (NCO) without reciprocal exchange resulting from synthesis-dependent strand annealing. In most eukaryotes, meiotic COs result from two pathways characterized as class I and class II (7). Class I COs depend on a group of proteins called ZMM (Zip1–4, Msh4–5, Mer3; first identified in budding yeast), which stabilizes recombination intermediates to promote class I CO formation (8). The class II CO pathway requires activity of structure-specific endonucleases including MUS81. In Arabidopsis, class I contributes to 85–90% of COs, whereas MUS81-dependent COs constitute a minor fraction (9,10).

Central to HR repair is the homologous recognition and DNA strand exchange of ssDNA overhangs catalyzed by two recombinases: (i) RAD51, which acts during mitosis and meiosis and (ii) DMC1, a meiosis-specific paralog present in most eukaryotes (11). The reason why many organisms have two recombinases and others only one remains unclear. Both recombinases can polymerize at the ssDNA overhangs to produce nucleoprotein filaments. RAD51- and DMC1-filaments forming nuclear foci localized on the chromosome are indicative of HR repair events in somatic and in meiotic cells (11,12). However, differences may lie in their relative contributions that appear to influence the fate of the repair event during meiosis. DMC1 is the main catalytic recombinase that promotes meiotic DSB repair on the homologous chromosome, likely to ensure CO formation among homologs. In contrast, RAD51 acts as an essential co-factor for DMC1-mediated repair (13,14). In somatic cells, RAD51, but not DMC1, is indispensable for HR repair. RAD51-ssDNA and DMC1-ssDNA filaments can be highly toxic intermediates if they remain unresolved in the cell. The DNA transactions mediated through these filaments appear dynamic and may be lost with defective filament assembly or impaired filament disassembly. To promote efficient HR repair and to avoid dead-end nucleoprotein complexes, a dynamic balance between their loading and unloading at break sites is achieved with pathway reversibility, implemented via positive and negative regulators (1). However, the interplay between multiple positive and negative regulators to control the dynamic assembly/disassembly of RAD51/DMC1 filaments in multicellular eukaryotes remains poorly characterized.

BRCA2 (breast cancer susceptibility protein 2), a highly conserved protein in multicellular eukaryotes, is a positive mediator protein of RAD51 and DMC1 nucleation onto ssDNA overhangs as shown in *in vitro* studies (15,16). BRCA2 interacts directly with RAD51 and DMC1 (16–19). The current model supports the notion of BRCA2 being a cargo to recruit RAD51 and DMC1 molecules on ssDNA by virtue of its binding affinity for ssDNA and its capacity to displace the replication protein A (RPA2) coated on ssDNA overhangs. These biochemical studies are well supported, with *in vivo* roles of BRCA2 regulating RAD51- and DMC1-mediated HR. *BRCA2*-deficient cells show hypersensitivity to DNA damaging agents and exhibit gross genomic instability (20–22). Further, the disruption of BRCA2 function leads to aberrant meiosis as well. In Arabidopsis, there are two *BRCA2* homologs: *BRCA2A* and *BRCA2B*. The Arabidopsis *brca2a brca2b* double mutant is sterile with defective meiosis showing no RAD51 and DMC1 foci, no synapsis and unrepaired meiotic breaks resulting in chromosome fragmentation (21,23). Arabidopsis *brca2a* and *brca2b* single mutants are however fertile with normal meiosis, suggesting that the two paralogs have redundant roles during meiosis. However, *brca2a*, but not *brca2b*, is hypersensitive to DNA damage, revealing functional differences between BRCA2A and BRCA2B for somatic DNA repair (20). These *BRCA2* deficiency phenotypes raise a question as to why RAD51 and DMC1 fail to form filaments *in vivo*, although they are capable of doing so *in vitro* in the absence of BRCA2 (24). It is therefore conceivable that active mechanisms prevent RAD51 and DMC1 from forming filaments *in vivo*.

We recently identified a novel FIGL1 complex comprising FIDGETIN-LIKE-1 (FIGL1) and FIDGETIN-LIKE-1 INTERACTING PROTEIN (FLIP) that likely acts as a negative regulator of RAD51 and DMC1 during meiosis. Mutations in *FIGL1* and *FLIP* in *Arabidopsis thaliana* increase meiotic CO frequency and modulate the number and/or dynamics of RAD51/DMC1 foci (25,26). The widely conserved FIGL1 complex physically interacts with RAD51 and DMC1. FIGL1 is also required for efficient HR in human somatic cells, attributed to its interaction with RAD51 (27). Overall, the FIGL1 complex appears to be a conserved regulator of recombinase activity at the strand invasion step of HR, in somatic and in meiotic cells. FIGL1 belongs to the large family of AAA-ATPase proteins that are implicated in structural remodeling, unfolding and disassembly of proteins or oligomer complexes (28,29).

In this study, we report a genetic interaction between BRCA2 and the anti-crossover factor FIGL1 and show that their antagonistic functions regulate RAD51 and DMC1 focus formation in both mitotic and meiotic cells, thereby promoting HR repair. Our data reveal a new regulatory step controlling the dynamics of RAD51 and DMC1 focus formation with the antagonistic functions of BRCA2 being a positive accelerator and FIGL1 hindering the reaction as negative regulator.

## MATERIALS AND METHODS

### Genetic material

The Arabidopsis plants were cultivated in a greenhouse as previously described (30). The T-DNA insertion and ethyl methane sulfonate (EMS) mutant lines used in this study were *brca2a-1* (N427769), *brca2b* (N537617) (21), *mus81* (N607515) (31), *figl1, figl1 mus81* (25), *zip4* (N568052) (32). The suppressor *figl1mus81(s)12/brca2a-2* line was sequenced using IIlumina technology at the Genome Analysis Centre, Norwich, UK. Mutations were identified in the MutDetect pipeline (33). Genetic mapping of causal mutations was performed in F2 fertile plants generated after backcross with *figl1 mus81*. Using polymerase chain reaction, we followed the segregation of homozygous EMS-generated mutations to identify a recessive mutation associated with partial restoration of fertility. The *brca2a-2* causal mutation was a C-to-T substitution at the TAI10 chr4:5656 position in *At4g00020*. The primers used for genotyping are listed in the supplemental_dataset file. Fertility of plants was examined by counting seeds per silique (fruit) on the scanned image of siliques fixed in 70% ethanol using Zeiss Zen software.

### Cytology techniques

Surface spreads of meiotic chromosomes from pollen mother cells were made and visualized with DAPI staining as previously described (34). For cytological detection of meiotic proteins, male meiotic chromosome spreads from prophase I were prepared as described in Armstrong *et al.* (35). Spread slides were immediately used for immunostaining of chromosome axis protein ASY1 or meiosis-specific cohesin REC8 antibodies to mark prophase I. Primary antibodies used for immunostaining were rabbit anti-DMC1 (1:20) (30), rat anti-RAD51 (1:50) (36), rabbit anti-REC8 (37) or rabbit (1:500) and guinea pig anti-ASY1 (1:250) (35). Secondary antibodies included Alexa fluor®488 (A-11006); Alexa fluor®568 (A-11011); Southern Biotech, Alexa fluor®568 (A11075) and super clonal anti-rabbit Alexa fluor®488, (A-27034) obtained from Thermo Fisher Scientific and were used in a 1:400 dilution. Images were obtained using a Zeiss AxioObserver microscope and were analyzed using Zeiss Zen software. DMC1 and RAD51 signals on meiotic chromosome spreads were acquired at 2 s exposure.

For RAD51 immunostaining, root tip nuclei were treated as previously described (38). Briefly, 5-day-old seedlings of each genotypes were incubated with or without 4 μg/ml mitomycin C (MMC) for 2 h to induce DNA damage. After briefly rinsing, root tips were fixed for 45 min in 4% paraformaldehyde in PME [50 mM piperazine-N,N'-bis(2-ethanesulphonic acid) (PIPES), pH 6.9; 5 mM MgSO4; 1 mM EGTA] followed by three washes in PME for 5 min. Then, root tips were digested for 30 min in 1% (w/v) cellulase, 0.5% (w/v) cytohelicase, 1% (w/v) pectolyase in PME and were washed three times for 5 min in PME. Digested cells were then squashed gently onto slides with a coverslip and immersed in liquid nitrogen. Slides were air-dried and stored at −80°C. RAD51 immunostaining with rat anti-RAD51 (36) antibodies, along with DAPI on the root-tip-squashed cells, was performed according to Da Ines *et al.* (12). Images were obtained using the z-stack option with a Zeiss AxioObserver microscope and were analyzed using Zeiss Zen software. RAD51 foci were counted manually on maximum intensity projection images generated from the z-stack. Histograms and statistical analysis were performed using GraphPad Prism 7 software.

### Root growth assays

To perform the root growth analysis, seeds from wild type, *figl1, brca2a-2 brca2b* and *figl1 brca2a-2 brca2b* lines were surface-sterilized and sown on solid medium containing 0.5 X Murashige and Skoog salts, 1% sucrose, 0.8% agar and 0, 0.5, 1.0, 2.0 or 4.0 μg/ml mitomycin C (Duchefa). After stratification for 2 days at 4°C, plants were grown in long-day conditions by keeping plates in a vertical position for 14 days. Plates were scanned and the primary root length of seedlings was measured using Fiji with the smart root plugin. Mean values calculated from the root length value from 10 to 20 seedlings for each genotype were plotted to examine MMC sensitivity. Reduced mean root length compared to wild-type was considered as sensitivity to MMC. Statistical significance was computed using a one-way ANOVA and Dunnett’s multiple-comparisons test.

## RESULTS

### A genetic screen identifies a mutation in Arabidopsis *BRC2A* that suppresses *figl1* phenotypes

We identified *FIGL1* as an anti-CO gene in a previous suppressor screen using Arabidopsis *zmm* mutants (25). Here, we set out to find antagonists of *FIGL1* to better understand its function and the regulation of recombination. The Arabidopsis *figl1* mutant is however fertile with no macroscopic phenotypes that could be easily screened to identify suppressors. Thus, we performed a suppressor screen using a *figl1 mus81* double mutant that displayed reduced fertility with short fruit length and low seed set. The reduced fertility in *figl1 mus81* was attributed to defective DSB repair due to persistent unrepaired breaks and chromosome fragmentation at metaphase I (25). Given that the loss of *FIGL1* and *MUS81* activity results in the accumulation of aberrant recombination intermediates, we sought to identify mutations in pro-DSB repair factors, which may antagonize *FIGL1, MUS81* or both and could enhance seed set of *figl1 mus81*. Thus, we applied EMS mutagenesis on *figl1 mus81* mutants, and screened the obtained population for enhanced fertility (longer fruits) as a proxy for an attenuated chromosome fragmentation defect at meiosis.

**Figure 1.**
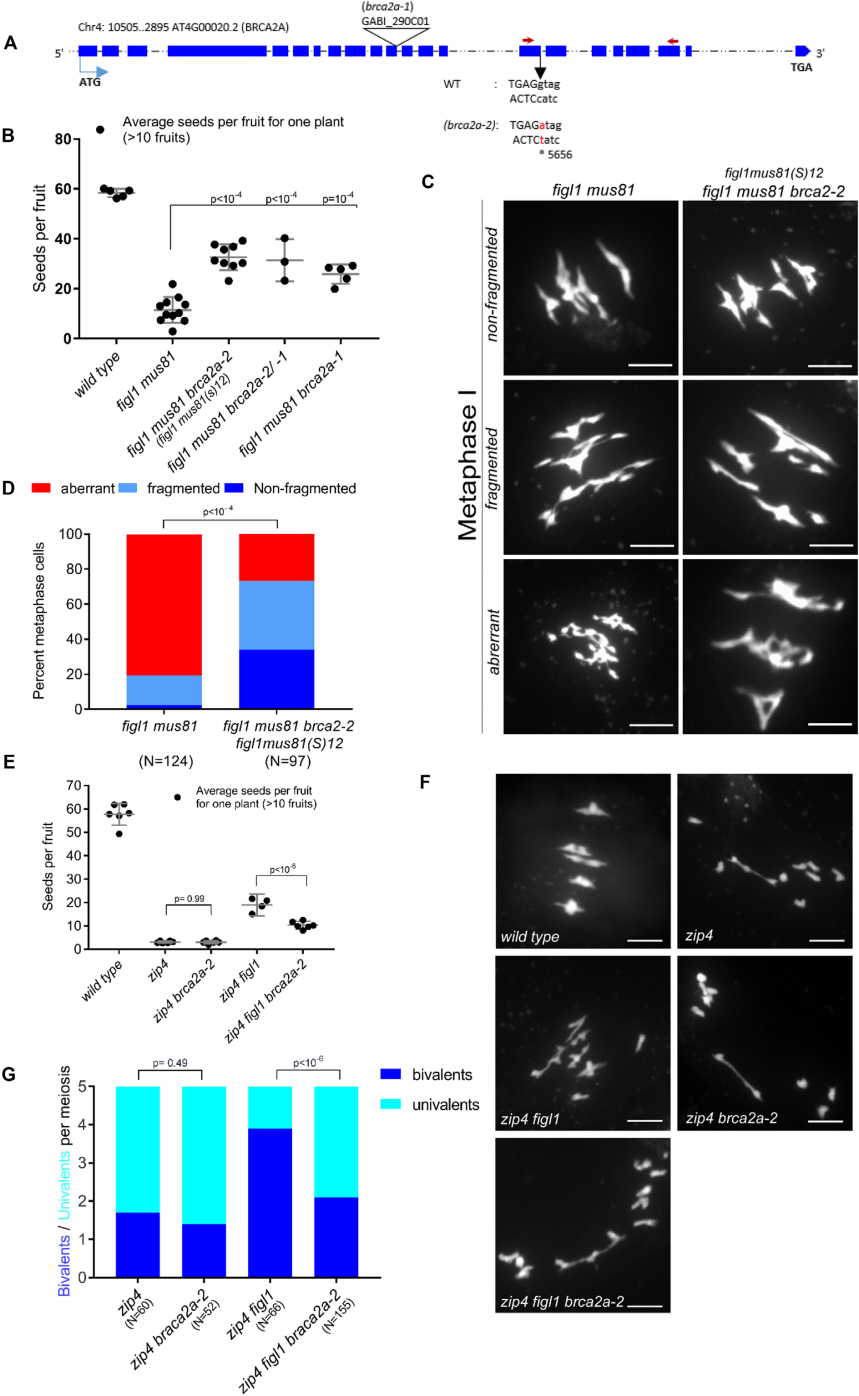
Mutation in *BRCA2A* suppresses *Figl1* phenotypes. (**A**) Schematic representation of the Arabidopsis *BRCA2A* gene with exons (in blue) along with start and stop codons. Positions of T-DNA insertion (*brca2a-1*) and an EMS-generated mutation (*brca2a-2*) at chromosome 4: 5656 that changes g/c to a/t (red) at the splice donor site of exon 15 in *Brca2a* are indicated. Arrows show the position of primers used for the *Brca2a* expression analysis in Supplementary Figure S1A. (**B**) Comparison of fertility based on the number of seeds per fruit in wild-type, *figl1 mus81, figl1 mus81 brca2a-2, figl1 mus81 brca2a-2/ -1* and *figl1 mus81 brca2a-2* plants. Each dot represents the average seeds per fruit for one plant, obtained by counting at least 10 fruits per plant. Shown *P*-values comparing different genotypes were computed by using Dunnett’s multiple-comparison test. Means along with standard deviation are shown in gray for each genotype. (**C**) DAPI-stained chromosome spread of male meiocytes showing three different classes of metaphase I from the *figl1 mus81* line and the suppressor *figl1 mus81 brca2a-2 / figl1mus81(S)12)* line; scale bars: 5 μm. (**D**) A comparison of non-fragmented (dark blue), fragmented (light blue) and aberrant (red) metaphase I from *figl1 mus81* and *figl1 mus81 brca2a-2 (figl1mus81(S)12)* mutants. *N* is the total number of metaphases analyzed for each genotype. *P*-values comparing the two genotypes were computed using the chi-square test. (**E**) Comparison of fertility by counting the number of seeds per fruit in wild-type, *zip4, zip4 brca2a-2, zip4 figl1* and *zip4 figl1 brca2a-2* plants. Each dot represents the average number of seeds per fruit for one plant, obtained by counting at least 10 fruits per plant. *P*-values comparing selected genotypes in pair were obtained from Sidak’s multiple-comparisons test. Means and standard deviations are shown in gray for each genotype. (**F**) Chromosome spread of male meiocytes at metaphase I stained with DAPI from wild-type, *zip4, zip4 figl1, zip4 brca2a-2* and *zip4 figl1 brca2a-2* plants; scale bars: 5 μm. (**G**) Comparing the average number of bivalents (blue) and pairs of univalent (turquoise) from *zip4, zip4 figl1, zip4 brca2a-2* and *zip4 figl1 brca2a-2* plants. *N* is the total number of metaphases analyzed for each genotype. *P*-values comparing bivalents from selected pairs of genotypes are computed from the Sidak’s multiple comparisons test.

We isolated a *figl1mus81(S)12* suppressor showing higher seed set compared with *figl1 mus81* (Figure 1B). This seed set however was still low compared with the wild-type, suggesting only partial restoration of fertility in the suppressor line. We next examined if the partial restoration of fertility could be due to attenuation of chromosome fragmentation at metaphase I, which would suggest restored DSB repair efficiency. Based on DAPI (4′,6-diamidino-2-phenylindole dihydrochloride) staining, we classified metaphases into three categories: (i) cells with five distinguishable DAPI bodies, suggesting non-fragmented chromosomes with almost wild-type-like DSB repair; (ii) fragmented metaphases with more than five DAPI bodies, considered as partially defective DSB repair; (iii) aberrant metaphases with entangled chromosome structures indicative of severe defective DSB repair. *figl1mus81(S)12* exhibited attenuated defects compared with *figl1 mus81* (Figure 1C and D), suggesting partial restoration of DSB repair. Whole genome sequencing and genetic mapping of *figl1mus81(S)12* identified the causal mutation at the splice donor site in exon 15 (chromosome 4: 5656, g to a) in the *AT4G00020* gene encoding for BRCA2A (*brca2a-2*) (Figure 1A). This mis-splicing results in the loss of 37 bp from exon 15 (Supplementary Figure S1A), leading to out-of-reading-frame translation after the E861 residue (BRCA2A-E861SS) that likely produces a BRCA2A protein truncated at the C-terminal end (Supplementary Figure S1B). We tested another mutated allele of *BRAC2A* (T-DNA insertion; GABI_290C04/ *brca2a-1*) (Figure 1A) that was also able to enhance fertility of *figl1 mus81* (Figure 1B). Further, *figl1 mus81 brca2a-2/brca2a-1* exhibited a higher seed set rate per fruit compared with *figl1 mus81* (Figure 1B), demonstrating that *brca2a-2* and *brca2a-1* were allelic. Mutations in *BRCA2A* can thus restore fertility and meiotic DNA repair of *figl1 mus81*.

We then examined if mutations in *BRCA2A* can suppress *figl1* phenotypes, also in the presence of *MUS81* activity. One feature of the *figl1* mutation is to restore bivalent/CO formation and fertility in *zip4* mutants (25). We therefore predicted that, if *BRCA2A* antagonizes *FIGL1* activity, the *brca2a-2* mutation would specifically reduce bivalent/CO formation and fertility of *figl1 zip4*. Indeed, *zip4 figl1 brca2a-2* showed a clear decrease in fertility compared with *zip4 figl1* (Figure 1E). Further, there was a significant decrease in bivalent formation in *zip4 figl1 brca2a-2* compared with *zip4 figl1* (Figure 1F and G), showing that mutation in *BRCA2A* reduces bivalent formation in the *zip4 figl1* background. The bivalent formation in *zip4* was not different in *zip4 brca2a-2*, suggesting that *brca2a-2* does not affect *zip4* per se. Taken together; our data demonstrate that *BRCA2A* can antagonize *FIGL1* activity.

### Epistasis analysis of *BRCA2A, BRCA2B* and *FIGL*1

**Figure 2.**
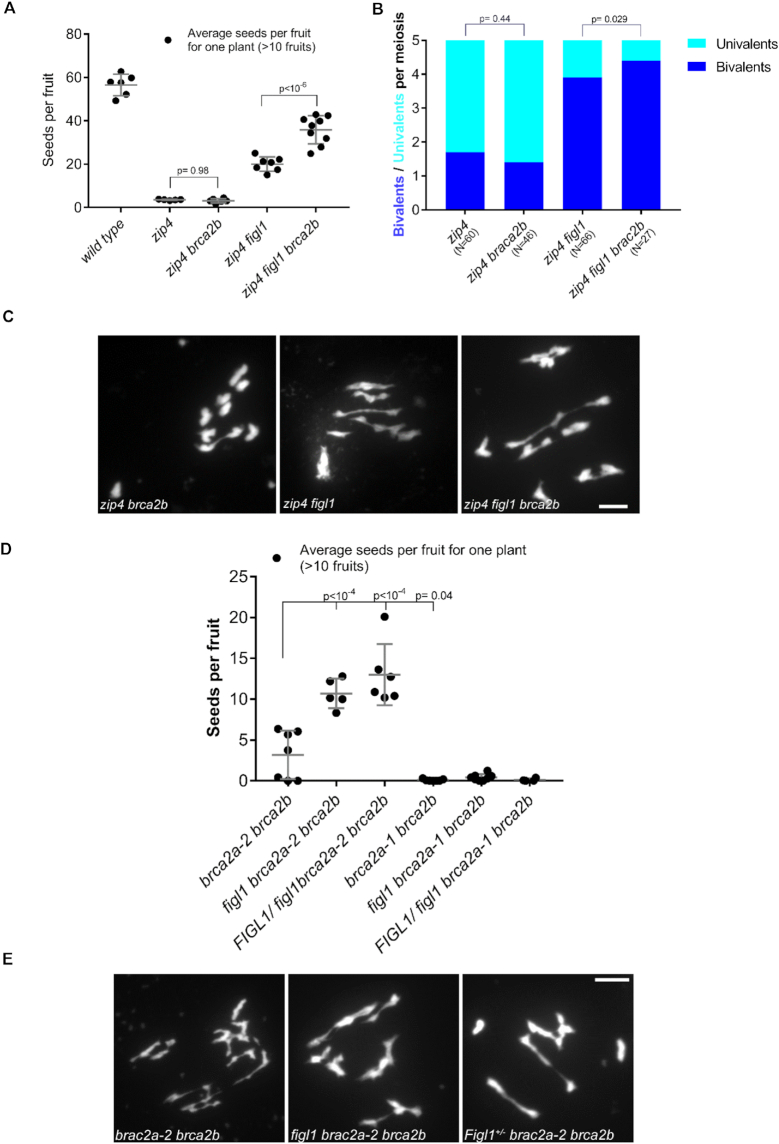
Epistasis analysis of *BRCA2A, BRCA2B* and *FIGL1*. (**A**) *brca2b*, a homolog of *Brca2a*, does not suppress zip4 *figl1*, as shown by fertility analysis in wild-type, *zip4, zip4 brca2b, zip4 figl1* and *zip4 figl1 brca2b* plants. Each dot indicates the average number of seeds per fruit for one plant, obtained by counting at least 10 fruits. *P*-values measuring significance between selected genotypes were computed from Sidak’s multiple-comparison test. Means and standard deviations are given in gray for each genotype. (**B**) Counting of bivalents (blue) and pairs of univalent (turquoise) in *zip4, zip4 brca2b, zip4 figl1* and *zip4 figl1 brca2b* plants.*N* is the total number of metaphases analyzed for each genotype. *P*-values comparing bivalent formation were computed using Sidak’s multiple-comparison test. (**C**) DAPI-stained chromosome spreads of male meiotic cells at metaphase I from *zip4 brca2b, zip4 figl1* and *zip4 figl1 brca2b* plants; scale bars: 5 μm. (**D**) Comparing fertility of plants with two different mutant *BRCA2A* alleles: *brca2a-2 brca2b, figl1 brca2a-2 brca2b, Figl1/figl1 brca2a-2 brca2b, brca2a-1 brca2b, figl1 brca2a-1 brca2b* and *Figl1/figl1 brca2a-1 brca2b*. Each dot indicates the average number of seeds per fruit for one plant, obtained by counting at least 10 fruits per plant. Dunnett’s multiple comparison test was used to obtain *P*-values, showing comparison of different genotypes. Means and standard deviations are given in gray for each genotype. (**E**) Chromosome spreads stained with DAPI of male meiocytes at metaphase I from *brca2a-2 brca2b, figl1 brca2a-2 brca2b* and *Figl1/ figl1 brca2a-2 brca2b* plants; scale bars: 5 μm.

BRCA2B is the second *BRCA2* homolog in Arabidopsis (Supplementary Figure S1C). A *brca2b* mutant was previously described as a likely null allele (21). The single *brca2a-1* and *brca2b* mutants are fertile without meiotic defects (Supplementary Figure S1D), whereas the *brca2a-1 brca2b* double mutant is fully sterile (21). We then tested if *BRCA2B* is also able to antagonize *FIGL1* functions as did *BRCA2A*. The fertility in *zip4 figl1 brca2b* was not reduced compared with *zip4 figl1* (Figure 2A). Further, the number of bivalent formations at metaphase was not reduced in *zip4 figl1 brca2b* compared with *zip4 figl1* (Figure 2B and C). *zip4 brca2b* plants did not show any reduction in fertility either, or in bivalent formation compared with *zip4*, suggesting no effect of *brac2b* mutation in *zip4* (Figure 2A and B). We concluded that mutations in *BRCA2B* do not suppress *figl1* phenotypes, implying a difference in either nature or importance of the *BRCA2A* and *BRCA2B* functions at meiosis.

We then investigated the functional consequences of the loss of antagonism between functions of *FIGL1* and the two Arabidopsis homologs *BRCA2A* and *BRAC2B*. We first compared the point mutation *brca2a-2* with the T-DNA insertion allele *brca2a-1* (21) for fertility and meiosis. The single *brca2a-2* and *brca2a-1* mutants showed normal fertility and no differences in bivalent formation at meiosis (Supplementary Figure S1D). In contrast to single mutants, *brca2a-1 brca2b* had almost no seeds and *brca2a-2 brca2b* produced only a few seeds (Figure 2D). The slight difference in fertility between the two double mutants suggests that *brca2a-1* is a null allele whereas *brca2a-2* retains some residual activity and that the C-terminus (after residue E861) of BRCA2A, which contains an OB-fold domain (21,39) (Supplementary Figure S1B and C), is important but not absolutely essential for BRCA2 activity.

Next, we tested the ability of the *figl1* mutation to restore fertility in *brca2a brca2b* double mutants. *figl1 brca2a-2 brca2b* mutants revealed a higher seed set compared with that of *brca2a-2 brca2b*. This confirms the antagonism between BRCA2A and FIGL1 functions. Intriguingly, *FIGL1/ figl1 brca2a-2 brca2b* also displayed a higher seed set compared with that of the *brca2a-2 brca2b* mutant (Figure 2D), suggesting that DNA repair efficiency is very sensitive to FIGL1/BRCA2 dosage. In contrast, introducing the *figl1* mutation (either homozygous or heterozygous) in *brca2a-1 brca2b* did not improve its fertility, further suggesting the *brca2a-1* is a stronger allele than *brca2a-2*, and that the *figl1* mutation is not able to re-establish DNA repair in the complete absence of BRCA2A/B (Figure 2D).

We then explored the causes behind the restoration of fertility in *figl1 braca2a-2 braca2b. brca2a-2 brca2b* showed a complete lack of bivalent formation and severe chromosome fragmentation at metaphase I, as in *brca2a-1 brca2b* (21). Further, analysis of metaphase chromosomes revealed fragmentation in *figl1 brca2a-2 brca2b* and *FIGL1/ figl1 brca2a-2 brca2b*, suggesting that the loss of *FIGL1* does not restore complete DSB repair and bivalent formation in *brca2a-2 brca2b* mutants (Figure 2E). However, the increased fertility of *figl1 brca2a-2 brca2b* compared with *brca2a-2 brca2b* suggests that the repair defect is reduced, but not enough to be detected by our analysis of chromosome shapes.

### Mutation in *FIGL1* restores RAD51/DMC1 focus formation and synapsis in the *brca2a braca2b* double mutant at meiosis

**Figure 3.**
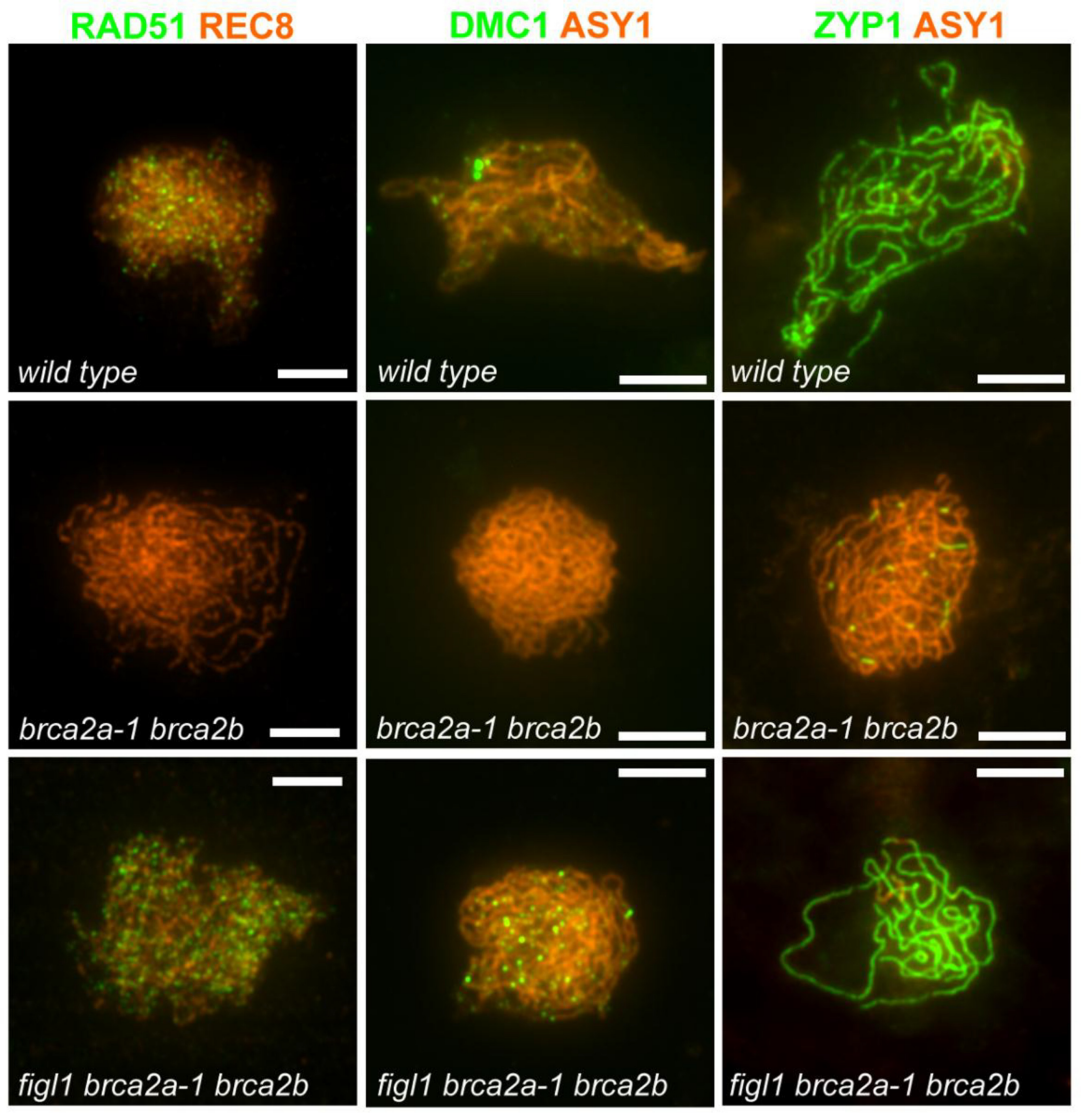
*Figl1* mutation in the *brca2a brca2b* double mutant background restores RAD51 and DMC1 focus formation and synapsis at meiosis. Double immunolocalization of RAD51 (green) with REC8 (orange) or DMC1 (green) in combination with ASY1 (orange) or ZYP1 (green) along with ASY1 (orange) are shown in merged images on surface-spread chromosomes of male meiocytes during prophase I in wild-type, *brca2a-1 brca2b* and *figl1 brca2a-1 brca2b* plants; scale bars: 5 μm.

RAD51 and DMC1 foci are localized on meiotic chromosomes as early recombination markers in the wild-type. Neither recombinase forms foci in *brca2a-1 brca2b* mutants, demonstrating the *in vivo* requirement for BRCA2 as a positive mediator (21). On the other hand, Arabidopsis *figl1* mutants showed an accumulation and/or delayed kinetics of RAD51 and DMC1 foci, suggesting that FIGL1 is a negative regulator of the recombinases (26). We hypothesized that RAD51 and DMC1 repair foci can be restored in triple mutants upon loss of *BRCA2* and *FIGL1* owing to their antagonistic properties. We thus performed immunolocalization of RAD51 and DMC1 on double (*brca2a-1 brca2b, brca2a-2 brca2b*) and triple (*figl1 brca2a-1 brca2b, figl1 brca2a-2 brca2b)* mutants in combination with staining of chromosome axis proteins, either REC8 or ASY1, to mark meiocytes in prophase I. We did not find any abnormalities in the localization of REC8 and ASY1 (Figure 3 and Supplementary Figure S2), suggesting no implication of BRCA2 and FIGL1 in forming chromosome axes. Meiocytes at prophase I from both *brca2a-1 brca2b* and *brca2a-2 brca2b* double mutants lacked RAD51 and DMC1 focus formation compared with wild-type meiocytes, consistent with the previous results obtained in the *brca2a-1 brca2b* background. In contrast, *figl1 brca2a-1 brca2b* and *figl1 brca2a-2 brca2b* triple-mutant meiocytes showed restored RAD51 and DMC1 focus formation in comparison with double-mutant meiocytes (Figure 3 and Supplementary Figure S2), demonstrating antagonistic functions of BRCA2 and FIGL1 in the regulation of RAD51/DMC1 focus formation. This result also suggests that BRACA2 is not essential *per se* for DMC1/RAD51 focus formation.

Further, *brca2a-1 brca2b* mutants show a complete lack of synapsis (19), a process in which ZYP1, a central element of the synaptonemal complex, polymerizes along the entire length of homologous chromosomes at the pachytene stage. This absence of synapsis is consistent with the fact that this process depends on the DMC1/RAD51-mediated strand invasion on homologs in Arabidopsis. We thus examined synapsis in the double (*brca2a-1 brca2b, brca2a-2 brca2b)* and the triple (*figl1 brca2a-1 brca2b, figl1 brca2a-2 brca2b)* mutants by performing double immunolocalization of ZYP1 and ASY1 on male meiocytes. Both the triple mutant *figl1 brca2a-1 brca2b* and *figl1 brca2a-2 brca2b* indeed showed restoration of synapsis through assembly of ZYP1 between homologs, compared with short stretches of ZYP1 observed in the double mutant *brca2a-1 brca2b* and *brca2a-2 brca2b* meiocytes (Figure 3 and Supplementary Figure S2). This restoration suggests that strand invasion can occur in the absence of both BRACA2 and FIGL1. Altogether, our data show that FIGL1 and BRCA2 antagonize/compete with each other to regulate RAD51/DMC1-dependent homology recognition during meiotic recombination.

### FIGL1 antagonizes BRCA2 activity to regulate RAD51 focus formation upon DNA damage in somatic cells

**Figure 4.**
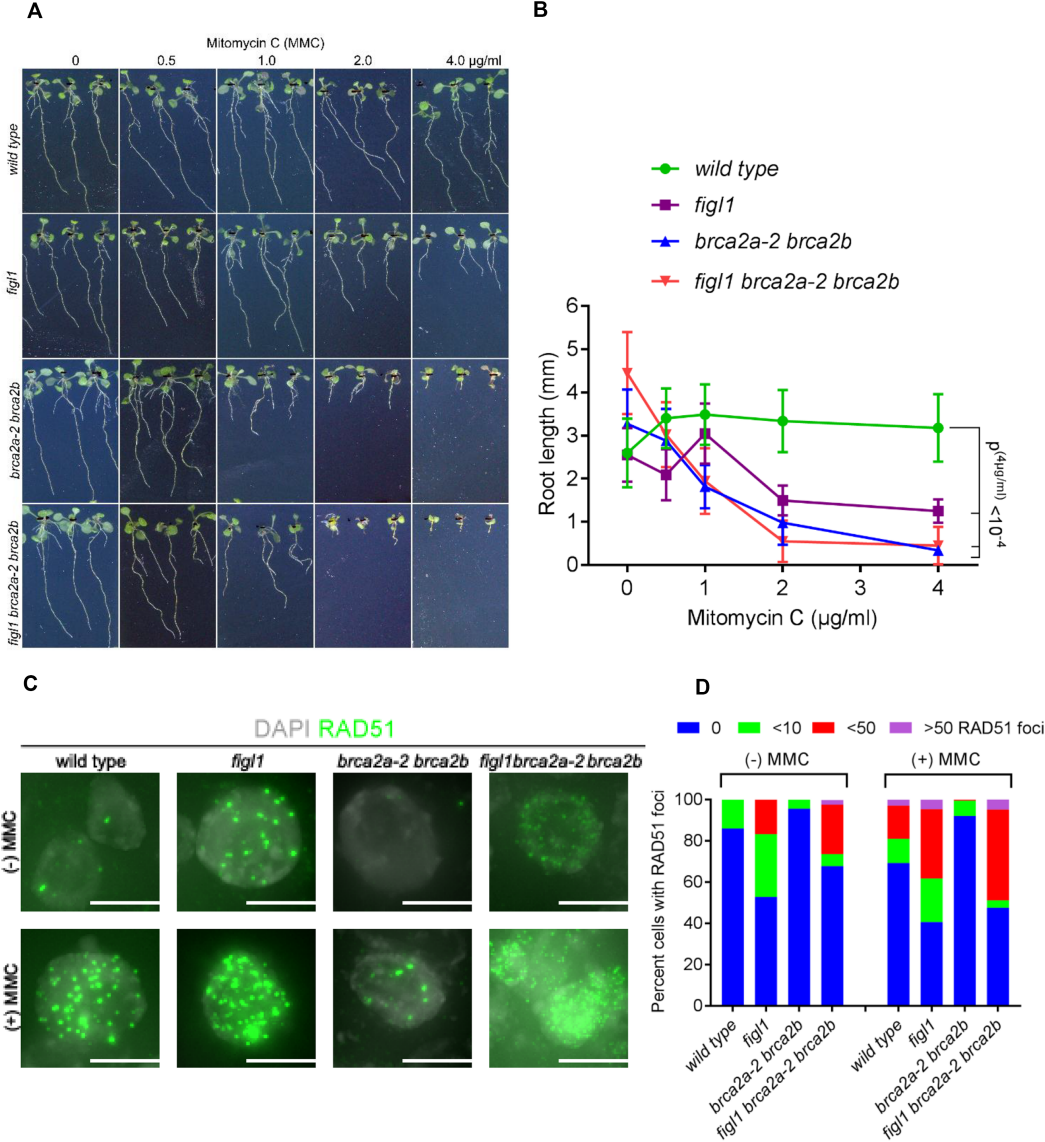
*figl1* mutation restores RAD51 focus formation in *braca2a braca2b* somatic cells, but does not improve mitomycin C (MMC) hypersensitivity. (**A**) *figl1, brca2a-2 brca2b* and *figl1 brca2a-2 brca2b* mutants are hypersensitive to MMC treatment. Root growth of plants germinated and grown on media containing 0, 0.5, 1.0, 2.0 or 4 μg/ml MMC. (**B**) Comparison of root growth length of wild-type, *figl1, brca2a-2 brca2b* and *figl1 brca2a-2 brca2b* mutant plants grown on different concentrations of MMC. Means and standard deviations calculated from 10 to 20 plants for each MMC concentration are plotted. Statistical significance was computed using Dunnett’s multiple-comparisons test. (**C**) Localization of RAD51 (green) in nuclei (DAPI, gray) by immunostaining the squashed root cells of wild-type and *figl1, brca2a-2 brca2b* and *figl1 brca2a-2 brca2b* mutant plants treated with 4 μg/ml MMC for 2 h. Merged images are shown. Scale bars: 5 μm. (**D**) Quantification of RAD51 foci in root cells of wild-type and *figl1, brca2a-2 brca2b* and *figl1 brca2a-2 brca2b* mutant plants. Percentage of cells (>100 cells per genotype) with 0, <10, <50 and >50 RAD51 foci is shown for each genotype with and without MMC treatment.

We next examined functional consequences of the loss of antagonism between BRCA2 and FIGL1 in somatic cells upon induction of DNA damage. Here, we tested whether *figl1, brca2a-2 brca2b* and *figl1 brca2a-2 brca2b* mutants are sensitive to MMC treatment. We thus performed root growth assays on media containing different MMC concentrations for each genotype and measured root length after 14 days of growth (Figure 4A). As shown in Figure 4B, we plotted mean values of root length (mm) ± SD to quantify the growth levels of each genotype. Wild-type plants showed no differences, with almost similar root growth on all tested MMC concentrations. The root growth of *brca2a-2 brca2b* compared with that of the wild-type at 2.0 and 4.0 μg/ml MMC was strongly affected, demonstrating the hypersensitivity of *brca2a-2 brca2b* (Figure 4A and B), as observed previously for *brca2a-1 brca2b*. Further, we noticed a significant reduction in root growth for *figl1* (Sidak’s multiple-comparison test, *P* < 0.000001) in comparison with the wild-type at concentrations of 2.0 and 4.0 μg/ml MMC (Figure 4A and B). This reduction in root growth suggests that FIGL1 is required for normal HR repair in mitotic cells upon DNA damage. Comparison of root growth between *figl1* and *brca2a-2 brca2b* plants also revealed that *figl1* mutants were less affected (Figure 4A and B). At those MMC concentrations, *figl1 brca2a-2 brca2b* plants did not show root growth different from *brca2a-2 brca2b*, but both exhibited yet stronger decreases in root growth in comparison with *figl1* plants (at 4 μg/ml MMC, Sidak’s multiple-comparison test, *P* < 0.00001) (Figure 4A and B). Altogether, our results demonstrate that *figl1* and *brca2a-2 brca2b* are hypersensitive to MMC treatment and that FIGL1 and BRCA2 play essential roles for repairing breaks arising from treatment of this DNA-crosslinking agent. However, loss of *FIGL1* in *brca2a-2 brca2b* does not help rescue root growth upon DNA damage, which contrasts with the ability of *figl1* to increase the fertility of the same genotype.

We then tested whether the MMC hypersensitivity of *figl1* and *brca2a-2 brca2b* mutants is due to a defective HR process leading to the presence of unrepaired breaks, monitored as RAD51 foci in root cells upon treatment. Using anti-RAD51 antibodies, we immunolocalized RAD51 in wild-type, *figl1, brca2a-2 brca2b* and *figl1 brca2a-2 brca2b* root cells without or with MMC treatment (4 μg/ml) for 2 h (Figure 4C and D; Supplementary Figure S3). To quantify RAD51 foci, we distributed root cells into four classes: (i) nuclei without any foci, (ii) nuclei with 1–10 foci, (iii) nuclei with 11–50 foci, (iv) nuclei with more than 50 foci. We recorded significantly lower numbers of total RAD51 positive nuclei in *brca2a-2 brca2b* compared with that of the wild-type with or without MMC treatment (Figure 4C and D). This strong reduction in RAD51 positive nuclei in *brca2a-2 brca2b* confirms that BRCA2 activity is important for RAD51 focus formation in somatic cells. Interestingly, *FIGL1*-deficient root cells exhibited a large increase in RAD51 foci in the absence and presence of MMC (Figure 4C and D). This suggests that FIGL1 negatively regulates RAD51 focus formation in somatic cells. Importantly, we found that *figl1* mutation restores formation of RAD51 foci in *brca2a-2 brca2b* nuclei with or without MMC treatment (Figure 4C and D). This restoration demonstrates that the antagonism between FIGL1 and BRCA2 also regulates RAD51 focus formation in somatic cells. Overall, these data support the positive function of BRCA2 and the negative function of FIGL1 in the regulation of RAD51 in somatic cells and their interplay is required to facilitate HR repair.

## DISCUSSION

Here, we identified a genetic interaction between Arabidopsis *BRCA2* and *FIGL1* that is essential for regulating HR repair in both mitotic and meiotic cells. We established that BRCA2 and FIGL1 antagonize each other for RAD51 focus formation in somatic cells, and for RAD51 and DMC1 focus formation and synapsis in meiotic cells.

We previously identified Arabidopsis FIGL1 as an anti-CO factor that limits meiotic crossovers. Our current hypothesis is that FIGL1 prevents formation of aberrant recombination intermediates in wild-type meiosis. We propose that FIGL1 negatively regulates the strand invasion step of HR through direct interaction with RAD51 and DMC1 (26). Absence of FIGL1 would thus result in exacerbated RAD51/DMC1 activity leading to the formation of aberrant recombination intermediates requiring the structure-specific endonuclease MUS81 to be repaired. We found that a mutation in *BRCA2A* attenuates meiotic DSB repair defects in *figl1 mus81*. We therefore suggest that the mutation of *BRCA2A* reduces BRCA2 dosage, presumably without affecting the levels of the second homolog BRCA2B. This reduction in BRCA2 dosage attenuates meiotic defects, suggesting that there may indeed be less aberrant recombination intermediates. BRCA2A and BRCA2B appear to have redundant activities as judged from normal fertility and meiosis in *brca2a-2* and *brca2b* single mutants. However, only the mutation of *BRCA2A*, but not of *BRCA2B*, restored bivalent formation in the *zip4 figl1* background. This observation suggests that BRCA2A functions are slightly different from those of BRCA2B, either in nature or dosage, for bivalent formation. Such differences in functionality between BRCA2A and BRCA2B has also been reported for somatic HR and may be due to differential expression levels (20). Importantly, we also noticed dosage effects for FIGL1 activity in *FIGL1/figl1 brca2a-2 brca2b* plants showing significantly higher numbers of seeds per fruit compared with *brca2a-2 brca2b*. Therefore, partial loss of FIGL1 activity in *brca2a-2 brca2b* may compensate for residual/reduced levels of BRCA2 to facilitate DSB repair. Altogether, dosage effects suggest that some critical threshold levels of both BRAC2 and FIGL1 are essential to promote accurate HR repair.

Our data indicate that antagonism between BRCA2 and FIGL1, operating in both mitotic and meiotic cells, regulates RAD51- and DMC1-dependent homology recognition and the strand invasion step during HR repair. Thus, how does antagonism between BRCA2 and FIGL1 modulate RAD51 and DMC1 focus formation? A key to maintaining genome stability via HR is to promote legitimate repair and, at the same time, restrict inappropriate repair. Activity of RAD51- and DMC1-filaments generates DNA transactions that lie at the intersection of multiple pathways to yield CO/NCO involving different molecular mechanisms within HR. These DNA transactions, at least during meiosis, appear to be highly dynamic and can generate a variety of recombination intermediates (40,41). We propose that the antagonistic regulation of RAD51- and DMC1-focus formation acts as a ‘stop-and-go’ mechanism, whereby FIGL1 applies the brakes by dismantling RAD51/DMC1 filaments and BRCA2 act as an accelerator that stimulates filament assembly. In this scenario, in the wild-type, BRCA2 promotes HR to push the reaction forward through nucleation and stabilization of filaments, and FIGL1 allows the pathway to reverse and restart, restricting HR that may originate from repair- and non-repair-associated DNA-bound forms of RAD51/DMC1. In turn, this mechanism may contribute to the dynamic nature of DNA transactions during HR.

The orthologs of both BRCA2 and FIGL1 are absent in yeast, suggesting that this antagonism is functionally mediated through other proteins. Our data are consistent with a proposed model in budding yeast wherein antagonism between the stabilizing function of RAD51 paralogs Rad55–Rad57 and the destabilizing function of Srs2 anti-recombinase modulates RAD51-filament/focus formation in somatic HR repair (42). In analogy to this model in Arabidopsis, BRCA2 provides the stabilizing functions and FIGL1 acts as an anti-recombinase to destabilize the filaments. *SRS2* also exists in Arabidopsis (43) and may also participate in this equilibrium. This model predicts that deletion of stabilizing or destabilizing activity shifts the balance in a backward or forward direction, respectively. Likewise, we observed that the equilibrium of RAD51/DMC1 foci was shifted toward low numbers of foci in *brca2a-2 brca2b*, whereas *figl1* mutants showed an increase in RAD51/DMC1 focus formation. Importantly, we also observed the restoration of RAD51/DMC1 focus formation in *figl1 brca2a-2 brca2b*. Based on these results, we propose that BRCA2 protects filaments from the likely anti-recombinase activity of FIGL1, in addition to its other known roles including *in vivo* nucleation of RAD51/DMC1 filaments. Whether BRCA2 does so by directly interacting with FIGL1, as does the Rad55–Rad57 complex to counteract Srs2 activity, remains to be determined. Alternatively, BRCA2 and FIGL1 act competitively to reach a dynamic equilibrium between assembly and disassembly of filaments. Further, the restoration of RAD51/DMC1 foci in *figl1 brca2a-2 brca2b* is insufficient to restore either wild-type level of fertility or normal root growth upon DNA damage induced by MMC treatment. This insufficiency suggests that the execution of complete HR repair requires the presence of both BRCA2 and FIGL1 activity. A number of observations are compatible with this interpretation. BRCA2 deficiency is hallmarked by gross genome instability in both somatic and meiotic cells (21,23,44). In somatic cells, BRCA2 prevents gross genomic instability by counteracting non-homologous end-joining pathways (45), which does not seem to be the case during meiosis (46). It is tempting to propose that FIGL1 inhibits HR repair in the absence of BRCA2 by dismantling RAD51/DMC1 filaments, which may result in unrepaired breaks or break repair via other pathways. Loss of FIGL1 activity can also induce genome instability. Although not observed in Arabidopsis, rice *figl1* mutants display severe meiotic chromosome fragmentation showing unrepaired breaks (47).

In conclusion, we revealed an interplay between BRCA2 and FIGL1 that appears to ensure accurate HR and inhibit illegitimate HR when required. This interplay is important for completing HR repair under different cellular contexts and may be of particular interest to advance our understanding on genomic instability mechanisms associated with human pathologies such as cancer.

## DATA AVAILABILITY

Supplemental_Dataset: A spreadsheet file with nine sheets named according to the corresponding figures contains the raw data along with the analysis and the calculation of statistical significance tests used. In an additional sheet, the list of oligonucleotides used in this study is given.

## Supplementary Material

gkz225_Supplemental_Files
