# Supplementary material for: Antagonism between BRCA2 and FIGL1 regulates homologous recombination
Source: Nucleic Acids Res. 2019 Apr 3;47(10):5170–80. doi: 10.1093/nar/gkz225 (PMC6547764; doi:10.1093/nar/gkz225)
Supplement: gkz225_Supplemental_Files [file gkz225_supplemental_files.zip › Brca2_SUPP_figures.pdf]

Figure S1.

A. Expression analysis shows detection of *BRCA2A* transcripts in wild type*, brac2a-2, brca2a-1 and brca2a-2 brca2b* 10-day-old seedlings with RT-PCR using *BRCA2A* specific primers. Sequencing of PCR products from respective mutants suggests an alternative splicing that results in the loss of 37 nucleotides from exon 15 in the *brca2a-2* mutant sequence (in red) aligned with the wild-type sequence (in blue). *Adenine phosphoribosyltransferase* (*APT*) gene expression was used as a positive control. (MW; molecular weight, kbp; kilobase pair).

B. The sequences of full-length (1151 aa) and a truncated form of BRCA2A (881 aa) are shown. Mutation at the splice donor site in the exon 15 results in alternatively spliced mRNA that likely produces a truncated BRCA2A protein due to out-of-reading-frame translation after E861 (red bold), leading to the loss of 270 aa (shown in red) at the C-terminus.

C. Organization of conserved protein domains (BRC repeats, PhePP motif, helical domain and oligonucleotide binding (OB) domains) in Arabidopsis BRCA2A (1151 aa) and BRCA2B (1155 aa). Alignment of BRCA2A and BRCA2B shows sequence identity (in blue) between both Arabidopsis paralogues.

D. Counting of ring- and rod-shaped bivalent chromosomes indicative of ≥2 or ≥1 crossovers per bivalent at metaphase I, respectively, in *brca2b*, *brca2a-1* and *brca2a-2* plants. Tukey's multiple comparison test shows no statistically significant differences in ring bivalent formation in *brca2b*, *brca2a-1* and *brca2a-2*.

Figure S2: Restoration of RAD51 and DMC1 focus formation and synapsis at meiosis in *figl1 brca2a-2 brca2b* mutants.

Double immunolocalization of RAD51 (green) with REC8 (orange), or DMC1 (green) in combination with ASY1 (orange) or ZYP1 (green) and ASY1 (orange) is shown in merged images on surface-spread chromosomes of male meiocytes during prophase I in wild type, *brca2a-2 brca2b* and *figl1 brca2a-2 brca2b* plants. Scale bars: 5 µm

Figure S3: Restoration of RAD51 focus formation in root cells in *figl1 brca2a-2 brca2b* mutants. Localization of RAD51 (green) in nuclei stained with DAPI (grey) along with merged images with >20 nuclei is shown by immunostaining on the squashed root cells of wild type, *figl1, brca2a-2 brca2b, and figl1 brca2a-2 brca2b* mutant plants left untreated or treated with 4 µg/ml MMC for 2 h. Scale bars: 5 µm

Figure S1

A

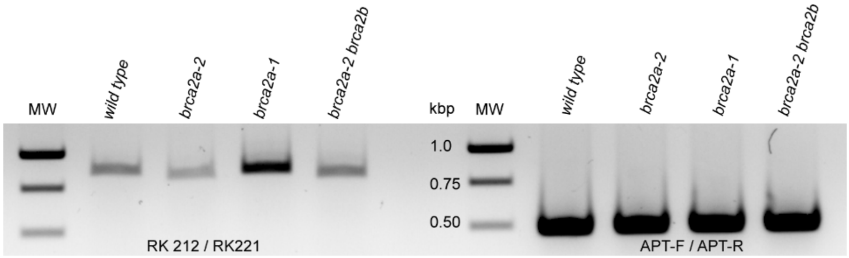


```
wild type ATTATACAGCTGCATAATCAGAGGCGCTCAGCTCTTGTTGAGGGTATTATGTGTGAGTAC
          ||||||||||||||||||||||||||||||||||||||||||||||||||||||||||||
brca2a-2  ATTATACAGCTGCATAATCAGAGGCGCTCAGCTCTTGTTGAGGGTATTATGTGTGAGTAC

wild type CAGAGAGGAATTAATGGTGTTCACAGCCAGAACGACACTGACAGCGAAGAAGGAGCCAAG
          ||||||||||||||||||||||||||||||||||||||||||||||||||||||||||||
brca2a-2  CAGAGAGGAATTAATGGTGTTCACAGCCAGAACGACACTGACAGCGAAGAAGGAGCCAAG

wild type ATCTTTAAGCTGTTAGAGACGGCTGCTGAACCTGAATTTCTTATGGCAGAAATGAGCCCA
          ||||||||||||||||||||||||||||||||||||||||||||||||||||||||||||
brca2a-2  ATCTTTAAGCTGTTAGAGACGGCTGCTGAACCTGAATTTCTTATGGCAGAAATGAGCCCA

wild type GAGCAGTTGAGATCTTTCACTACATATAAAGCAAAATTTGAGGCAGCCCAGCAAATGCGG
          ||||||                                     |||||||||||||||||
brca2a-2  GAGCAG-------------------------------------CAGCCCAGCAAATGCGG
```

B

BRCA2A: 1151 amino acids

MSTWQLFPDSSGDGFRWEVAGRILQSVSDSTPTKALESTAPLPSMADLLLQGCSKLIAREEAMPGEIPMFRTGLGKSVVL
KESSIAKAKSILAEKVTYSDLRNTNCSIPQMRQVDTAETLPMFRTASGKSVPLKESSIAKAMSILGSDKIIDSDNVLPRE
SGFGVSNSLFQTASNKKVNVSSAGLARAKALLGLEEDDLNGFNHVNQSSSSSQQHGWSGLKTHEEFDATVVKHHSGTPGQ
YEDYVSGKRSEVLNPSLKVPPTKFQTAGGKSLSVSAEALKRARNLLGDPELGSFFDDVAGGDQFFTPEKDERLSDIAINN
GSANRGYIAHEEKTSNKHTPNSFVSPLWSSSKQFSSVNLENLASGGNLIKKFDAAVDETDCALNATHGLSNNRSLASDMA
VNNSKVNGFIPRGRQPGRPADQPLVDITNRRDTAYAYNKQDSTQKKRLGKTVSVSPFKRPRISSFKTPSKKHALQASSGL
SVVSCDTLTSKKVLSTRYPEKSPRVYIKDFFGMHPTATTRMDYVPDHVRRIKSSNADKYVFCDESSSNKVGAETFLQMLA
ESGASLQHASRKWVTNHYRWIVWKLACYDIYYPAKCRGNFLTITNVLEELKYRYEREVNHGHCSAIKRILSGDAPASSMM
VLCISAINPKTDNDSQEAHCSDSCSNVKVELTDGWYSMNAALDVVLTKQLNAGKLFVGQKLRILGAGLSGWATPTSPLEA
VISSTICLLLNINGTYRAHWADRLGFCKEIGVPLALNCIKCNGGPVPKTLAGIKRIYPILYKERLGEKKSIVRSERIESR
IIQLHNQRRSALVEGIMCEYQRGINGVHSQNDTDSEEGAKIFKLLETAAEPEFLMAEMSP**E**QLRSFTTYKAKFEAAQQMR
KEKSVAETLEDAGLGERNVTPFMRIRLVGLTSLSYEGEHNPKEGIVTIWDPTERQRTELTEGKIYMMKGLVPINSDSEIL
YLHARGSSSRWQPLSPKDSENFQPFFNPRKPISLSNLGEIPLSSEFDIAAYVVYVGNAYTDVLQKKQWVFVTDGSAQHSG
EISNSLLAISFSTSFMDDSSVSHISHNLVGSVVGFCNLIKRAKDVTNEIWVAEAAENSVYFINAEAAYSSHLKTSSAHIQ
TWAKLSSSKSVIHELRQRVLSIIGACKSPSC*

BRCA2A^{E861SS} : 881 amino acids

MSTWQLFPDSSGDGFRWEVAGRILQSVSDSTPTKALESTAPLPSMADLLLQGCSKLIAREEAMPGEIPMFRTGLGKSVVL
KESSIAKAKSILAEKVTYSDLRNTNCSIPQMRQVDTAETLPMFRTASGKSVPLKESSIAKAMSILGSDKIIDSDNVLPRE
SGFGVSNSLFQTASNKKVNVSSAGLARAKALLGLEEDDLNGFNHVNQSSSSSQQHGWSGLKTHEEFDATVVKHHSGTPGQ
YEDYVSGKRSEVLNPSLKVPPTKFQTAGGKSLSVSAEALKRARNLLGDPELGSFFDDVAGGDQFFTPEKDERLSDIAINN
GSANRGYIAHEEKTSNKHTPNSFVSPLWSSSKQFSSVNLENLASGGNLIKKFDAAVDETDCALNATHGLSNNRSLASDMA
VNNSKVNGFIPRGRQPGRPADQPLVDITNRRDTAYAYNKQDSTQKKRLGKTVSVSPFKRPRISSFKTPSKKHALQASSGL
SVVSCDTLTSKKVLSTRYPEKSPRVYIKDFFGMHPTATTRMDYVPDHVRRIKSSNADKYVFCDESSSNKVGAETFLQMLA
ESGASLQHASRKWVTNHYRWIVWKLACYDIYYPAKCRGNFLTITNVLEELKYRYEREVNHGHCSAIKRILSGDAPASSMM
VLCISAINPKTDNDSQEAHCSDSCSNVKVELTDGWYSMNAALDVVLTKQLNAGKLFVGQKLRILGAGLSGWATPTSPLEA
VISSTICLLLNINGTYRAHWADRLGFCKEIGVPLALNCIKCNGGPVPKTLAGIKRIYPILYKERLGEKKSIVRSERIESR
IIQLHNQRRSALVEGIMCEYQRGINGVHSQNDTDSEEGAKIFKLLETAAEPEFLMAEMSP**E**QQPSKCGRKNQWQKLWKTL
V*

C

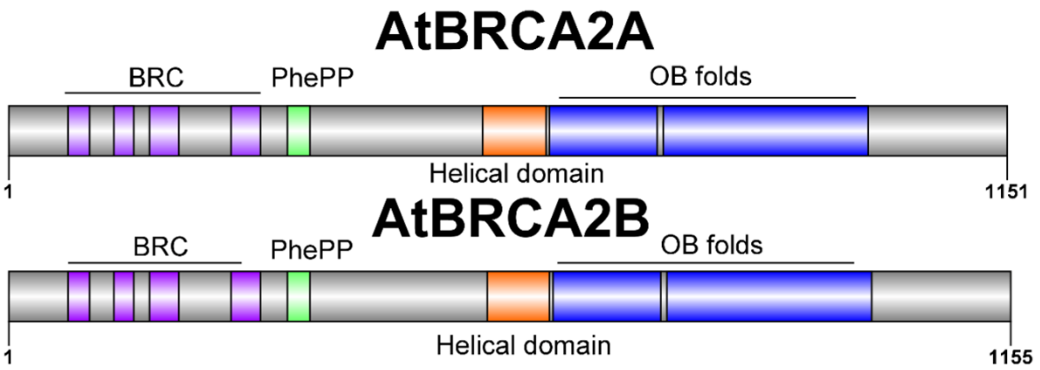
AtBRCA2A
BRC
PhePP
OB folds
Helical domain
1
1151
AtBRCA2B
BRC
PhePP
OB folds
Helical domain
1
1155

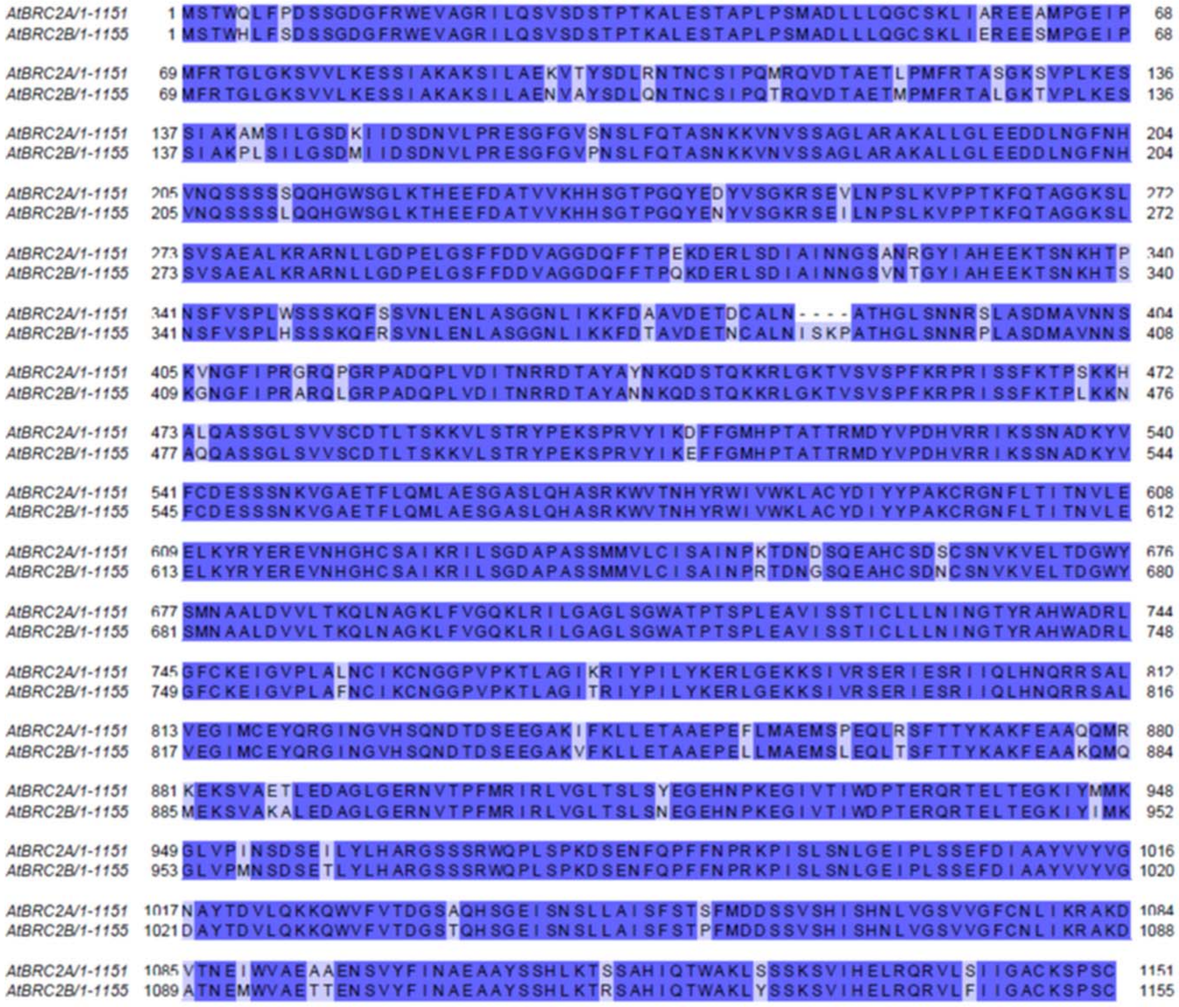
AtBRC2A/1-1151 1 MSTWQLFPDSSGDGFRWEVAGRILQSVSDSTPTKALESTAPLPSMADLLLQGCSKLIAREEAMPGEIP 68
AtBRC2B/1-1155 1 MSTWHLFSDSSGDGFRWEVAGRILQSVSDSTPTKALESTAPLPSMADLLLQGCSKLIEREESMPGEIP 68
AtBRC2A/1-1151 69 MFRTGLGKSVVLKESSIAKAKSILAEKVTYSDLRNTNCSIPQMRQVDTAETLPMFRTASGKSVPLKES 136
AtBRC2B/1-1155 69 MFRTGLGKSVVLKESSIAKAKSILAENVAYSDLQNTNCSIPQTRQVDTAETMPMFRTALGKTVPLKES 136
AtBRC2A/1-1151 137 SIAKAMSILGSDKIIDSDNVLPRESGFGVSNSLFQTASNKKVNVSSAGLARAKALLGLEEDDLNGFNH 204
AtBRC2B/1-1155 137 SIAKPLSILGSDMIIDSDNVLPRESGFGVPNSLFQTASNKKVNVSSAGLARAKALLGLEEDDLNGFNH 204
AtBRC2A/1-1151 205 VNQSSSSSQQHGWSGLKTHEEFDATVVKHHSGTPGQYEDYVSGKRSEVLNPSLKVPPTKFQTAGGKSL 272
AtBRC2B/1-1155 205 VNQSSSSLQQHGWSGLKTHEEFDATVVKHHSGTPGQYENYVSGKRSEILNPSLKVPPTKFQTAGGKSL 272
AtBRC2A/1-1151 273 SVSAEALKRARNLLGDPELGSFFDDVAGGDQFFTPEKDERLSDIAINNGSANRGYIAHEEKTSNKHTP 340
AtBRC2B/1-1155 273 SVSAEALKRARNLLGDPELGSFFDDVAGGDQFFTPQKDERLSDIAINNGSVNTGYIAHEEKTSNKHTS 340
AtBRC2A/1-1151 341 NSFVSPLWSSSKQFSSVNLENLASGGNLIKKFDAAVDETDCALN----ATHGLSNNRSLASDMAVNNS 404
AtBRC2B/1-1155 341 NSFVSPLHSSSKQFRSVNLENLASGGNLIKKFDTAVDETNCALNISKPATHGLSNNRPLASDMAVNNS 408
AtBRC2A/1-1151 405 KVNGFIPRGRQPGRPADQPLVDITNRRDTAYAYNKQDSTQKKRLGKTVSVSPFKRPRISSFKTPSKKH 472
AtBRC2B/1-1155 409 KGNGFIPRARQLGRPADQPLVDITNRRDTAYANNKQDSTQKKRLGKTVSVSPFKRPRISSFKTPLKKN 476
AtBRC2A/1-1151 473 ALQASSGLSVVSCDTLTSKKVLSTRYPEKSPRVYIKDFFGMHPTATTRMDYVPDHVRRIKSSNADKYV 540
AtBRC2B/1-1155 477 AQQASSGLSVVSCDTLTSKKVLSTRYPEKSPRVYIKEFFGMHPTATTRMDYVPDHVRRIKSSNADKYV 544
AtBRC2A/1-1151 541 FCDESSSNKVGAETFLQMLAESGASLQHASRKWVTNHYRWIVWKLACYDIYYPAKCRGNFLTITNVLE 608
AtBRC2B/1-1155 545 FCDESSSNKVGAETFLQMLAESGASLQHASRKWVTNHYRWIVWKLACYDIYYPAKCRGNFLTITNVLE 612
AtBRC2A/1-1151 609 ELKYRYEREVNHGHCSAIKRILSGDAPASSMMVLCISAINPKTDNDSQEAHCSDSCSNVKVELTDGWY 676
AtBRC2B/1-1155 613 ELKYRYEREVNHGHCSAIKRILSGDAPASSMMVLCISAINPRTDNGSQEAHCSDNCSNVKVELTDGWY 680
AtBRC2A/1-1151 677 SMNAALDVVLTKQLNAGKLFVGQKLRILGAGLSGWATPTSPLEAVISSTICLLLNINGTYRAHWADRL 744
AtBRC2B/1-1155 681 SMNAALDVVLTKQLNAGKLFVGQKLRILGAGLSGWATPTSPLEAVISSTICLLLNINGTYRAHWADRL 748
AtBRC2A/1-1151 745 GFCKEIGVPLALNCIKCNGGPVPKTLAGIKRIYPILYKERLGEKKSIVRSERIESRIIQLHNQRRSAL 812
AtBRC2B/1-1155 749 GFCKEIGVPLAFNCIKCNGGPVPKTLAGITRIYPILYKERLGEKKSIVRSERIESRIIQLHNQRRSAL 816
AtBRC2A/1-1151 813 VEGIMCEYQRGINGVHSQNDTDSEEGAKIFKLLETAAEPEFLMAEMSPEQLRSFTTYKAKFEAAQQMR 880
AtBRC2B/1-1155 817 VEGIMCEYQRGINGVHSQNDTDSEEGAKVFKLLETAAEPELLMAEMSLEQLTSFTTYKAKFEAAKQMQ 884
AtBRC2A/1-1151 881 KEKSVAETLEDAGLGERNVTPFMRIRLVGLTSLSYEGEHNPKEGIVTIWDPTERQRTELTEGKIYMMK 948
AtBRC2B/1-1155 885 MEKSVAKALEDAGLGERNVTPFMRIRLVGLTSLSNEGEHNPKEGIVTIWDPTERQRTELTEGKIYIMK 952
AtBRC2A/1-1151 949 GLVPINSDSEILYLHARGSSSRWQPLSPKDSENFQPFFNPRKPISLSNLGEIPLSSEFDIAAYVVYVG 1016
AtBRC2B/1-1155 953 GLVPMNSDSETLYLHARGSSSRWQPLSPKDSENFQPFFNPRKPISLSNLGEIPLSSEFDIAAYVVYVG 1020
AtBRC2A/1-1151 1017 NAYTDVLQKKQWVFVTDGSAQHSGEISNSLLAISFSTSFMDDSSVSHISHNLVGSVVGFCNLIKRAKD 1084
AtBRC2B/1-1155 1021 DAYTDVLQKKQWVFVTDGSTQHSGEISNSLLAISFSTPFMDDSSVSHISHNLVGSVVGFCNLIKRAKD 1088
AtBRC2A/1-1151 1085 VTNEIWVAEAAENSVYFINAEAAYSSHLKTSSAHIQTWAKLSSSKSVIHELRQRVLSIIGACKSPSC 1151
AtBRC2B/1-1155 1089 ATNEMWVAETTENSVYFINAEAAYSSHLKTRSAHIQTWAKLYSSKSVIHELRQRVLFIIGACKSPSC 1155

D

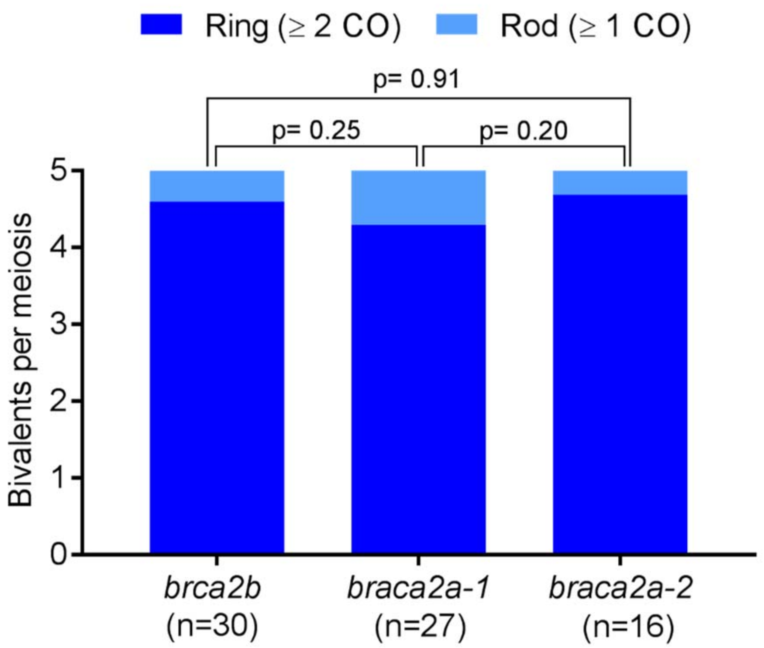

Ring (≥ 2 CO)
Rod (≥ 1 CO)
p= 0.91
p= 0.25
p= 0.20
Bivalents per meiosis
5
4
3
2
1
0
brca2b
(n=30)
braca2a-1
(n=27)
braca2a-2
(n=16)

Figure S2

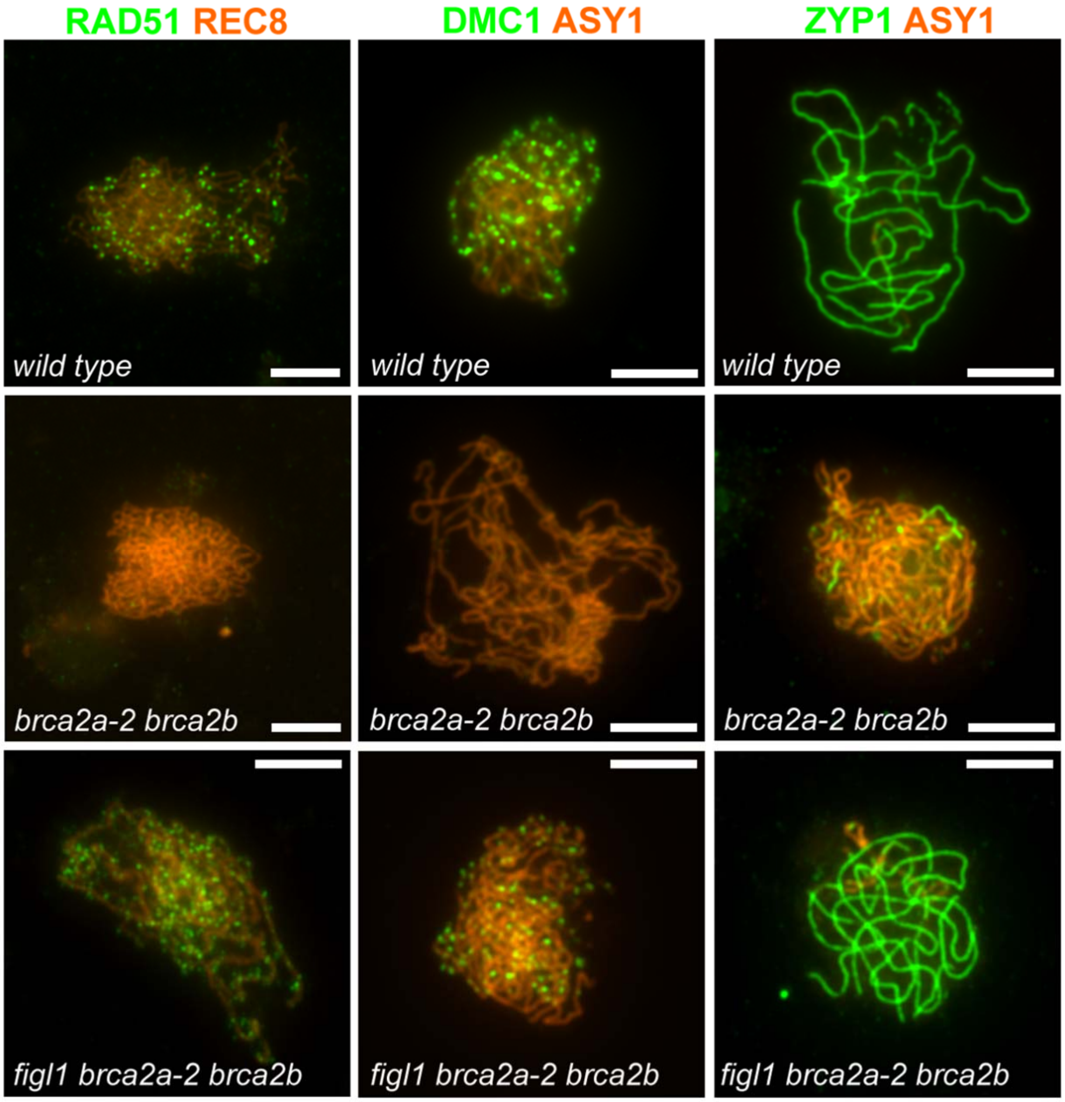

RAD51 REC8
DMC1 ASY1
ZYP1 ASY1
wild type
wild type
wild type
brca2a-2 brca2b
brca2a-2 brca2b
brca2a-2 brca2b
figl1 brca2a-2 brca2b
figl1 brca2a-2 brca2b
figl1 brca2a-2 brca2b

Figure S3

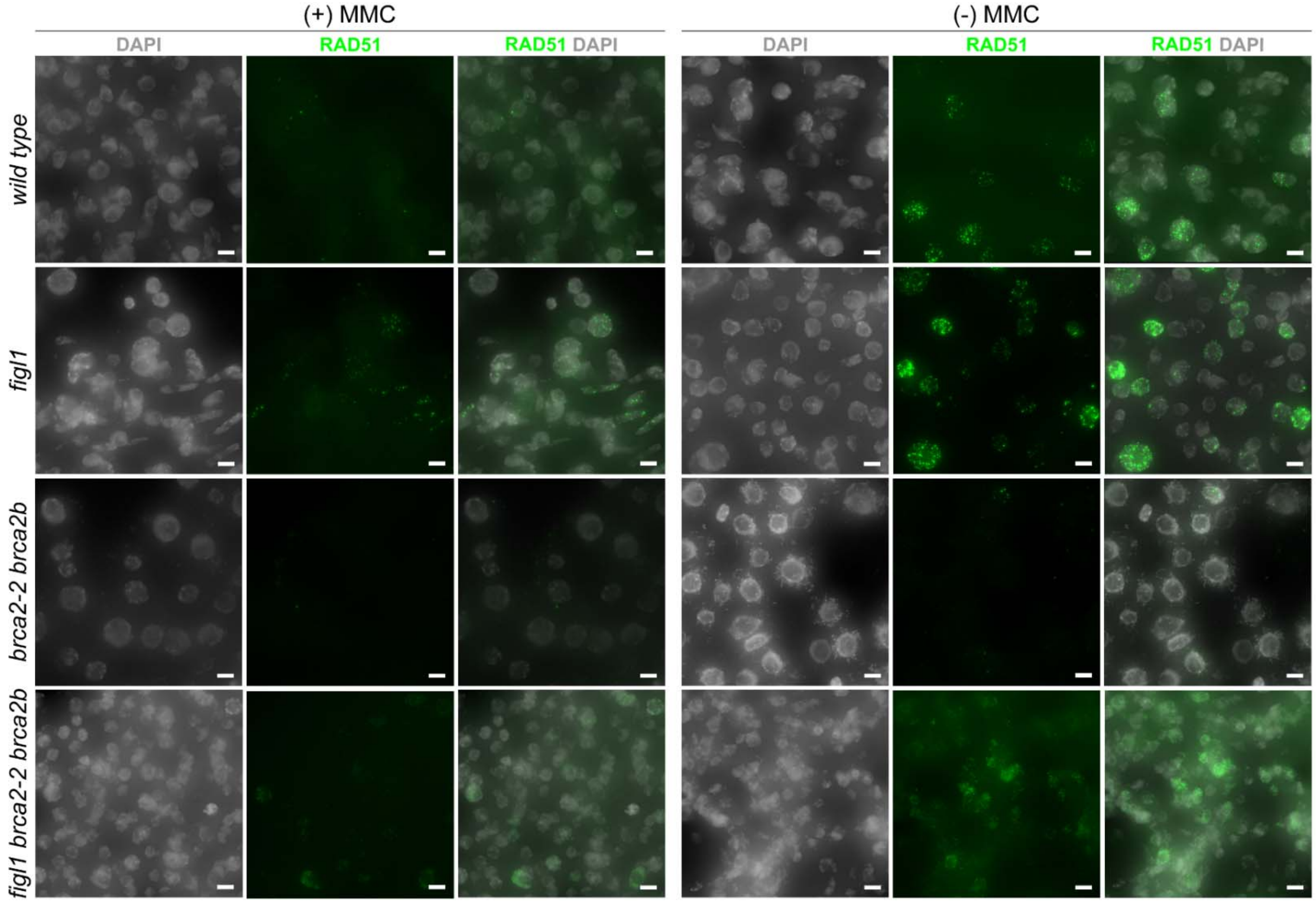

(+) MMC
(-) MMC
DAPI
RAD51
RAD51 DAPI
DAPI
RAD51
RAD51 DAPI
wild type
figl1
brca2-2 brca2b
figl1 brca2-2 brca2b
